# Multi-decadal trends in global terrestrial evapotranspiration and its components

**DOI:** 10.1038/srep19124

**Published:** 2016-01-11

**Authors:** Yongqiang Zhang, Jorge L. Peña-Arancibia, Tim R. McVicar, Francis H. S. Chiew, Jai Vaze, Changming Liu, Xingjie Lu, Hongxing Zheng, Yingping Wang, Yi Y. Liu, Diego G. Miralles, Ming Pan

**Affiliations:** 1 CSIRO Land and Water, GPO Box 1666, Canberra ACT 2601, Australia; 2 ARC Centre of Excellence for Climate System Science & Climate Change Research Centre, University of New South Wales, Sydney 2052, Australia; 3 Institute of Geographic Sciences and Natural Resources Research, Chinese Academy of Sciences, Beijing 100101, China; 4 CSIRO Ocean and Atmosphere, PMB #1, Aspendale, Victoria 3195, Australia; 5 Department of Earth Sciences, VU University Amsterdam, Amsterdam 1081 HV, The Netherlands; 6 Laboratory of Hydrology and Water Management, Department of Forest and Water Management, Ghent University, B-9000 Ghent, Belgium; 7 Department of Civil and Environmental Engineering, Princeton University, NJ 08544, USA

## Abstract

Evapotranspiration (ET) is the process by which liquid water becomes water vapor and energetically this accounts for much of incoming solar radiation. If this ET did not occur temperatures would be higher, so understanding ET trends is crucial to predict future temperatures. Recent studies have reported prolonged declines in ET in recent decades, although these declines may relate to climate variability. Here, we used a well-validated diagnostic model to estimate daily ET during 1981–2012, and its three components: transpiration from vegetation (E_t_), direct evaporation from the soil (E_s_) and vaporization of intercepted rainfall from vegetation (E_i_). During this period, ET over land has increased significantly (*p* < 0.01), caused by increases in E_t_ and E_i_, which are partially counteracted by E_s_ decreasing. These contrasting trends are primarily driven by increases in vegetation leaf area index, dominated by greening. The overall increase in E_t_ over land is about twofold of the decrease in E_s_. These opposing trends are not simulated by most Coupled Model Intercomparison Project phase 5 (CMIP5) models, and highlight the importance of realistically representing vegetation changes in earth system models for predicting future changes in the energy and water cycle.

Terrestrial ET is a key component of the energy and water cycles over global land^1^. It is the second largest component of the hydrological cycle after precipitation^2^,^3^. Variation of terrestrial ET influences precipitation^1^, and land surface water availability in water bodies, such as lakes and rivers. ET is the second largest component in the surface energy balance after net radiation^1^. Change in terrestrial ET, and its associated latent heat flux, will impact the sensible heat flux by changing land surface temperature, having important implications on regional and global warming^4^.

Recent studies have focused on ET trends in last several decades, and have attributed the ET trends to regional drought^1^, climate change^5^, or internal climate variability^6^. In addition to potential discrepancies in the direction and cause of these changes, the relative contribution of the three main components of ET (i.e., E_t_, E_s_ and E_i_) to these global trends remains unknown. ET components can respond differently to changes in environmental conditions and/or vegetation. For example, while E_t_ is dependent on plant phenology and water-use efficiency, E_s_ is mostly driven by the atmospheric demand for vapour, the availability of water in the soil, and the amount of vegetation above the soil, and E_i_ by the occurrence of rainfall and the characteristics of the vegetation stand.

Several studies report the partitioning of global land surface ET, mainly using global land models^7^ or isotope observations^8^,^9^. Although E_t_ is a major component of ET, the global ratio of E_t_ to ET remains uncertain^9^. More importantly, it is not clear how the ET components contribute to annual trend and variability in ET. To unravel the key mechanisms behind the trends in ET, and the contribution of each ET component to these trends over the last three decades, we used the observation-driven Penman-Monteith-Leuning (PML)^10^,^11^ model. The PML model has been chosen because of its sound physical basis, simple parameterization, and relatively straightforward application^10^,^11^,^12^; furthermore, it can be implemented globally, and at high spatial and temporal resolutions, when forced with readily-available gridded meteorological^13^,^14^,^15^ and satellite data^16^,^17^.

## Results

### Model validations

The PML estimated ET and its partitioning are comprehensively assessed from point to global scale, including: (i) catchment precipitation (P) and streamflow (Q) data; (ii) eddy-covariance flux tower data; (iii) satellite-derived soil moisture; (iv) field experiments of ET partitioning; (v) comparing annual E_i_/P ratio in homogeneous forests; (vi) evaluation of E_s_ in extreme climates; and (vii) model inter-comparison. This assessment includes five validations, being (i) to (v), and two evaluations, (vi) to (vii); all are outlined below in-turn.

**Figure 1:**
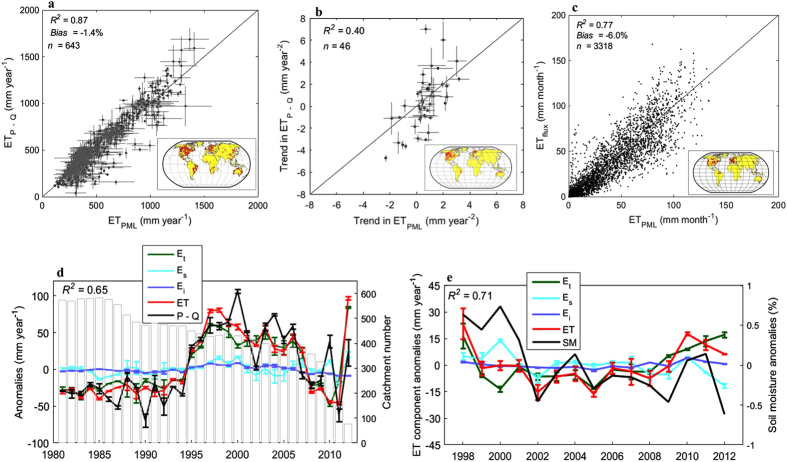
Validation of the PML-ET model. (**a**) Comparison of the estimated mean annual ET (1981–2012, mm year^−1^) to catchment ET (P–Q) observations in 643 catchments (red dots in the global map). (**b**) Comparison of estimated annual ET trend (mm year^−2^) to catchment annual ET (P–Q) for 46 large catchments (>10, 000 km^2^) with less than 3-year missing data. (**c**) Comparison of the estimated ET (mm month^−1^) to the measured ET at 95 flux sites (red dots in the global map). **(d**) Annual anomalies of the estimated ET, ET components, and catchment observed ET (P–Q), all aggregated from catchments (number of catchments per year provided using grey bars) with annual Q observations. (**e**) Annual anomalies of the estimated ET, ET components, and the observed microwave soil moisture, all aggregated between 40 °N and 40 °S (area covered by the microwave soil moisture data). Error bars for ET and its components are s.d. obtained from the two PML simulations. Error bars for P–Q are s.d. obtained from the two precipitation datasets. *R*^2^ in Fig. 1(**a–d)** is obtained from comparing measured ET and estimated ET. *R*^2^ in Fig. 1(**e)** is obtained from comparing soil moisture and estimated E_s_. The maps were generated using a commercial software MATLAB.

First, over a long-term catchment water balance, ‘observed’ mean annual ET is the difference between the observed catchment mean annual P–Q. The PML estimated ET compares well with long-term water-balance ET observations from 643 largely unregulated large (>2000 km^2^) catchments across the world (Fig. 1a), with coefficient of determination *R*^*2*^ = 0.87 and *Bias* = −1.4%. The time-series of aggregated estimated annual ET from the 643 catchments also show good correspondence to the annual P–Q aggregated series (Fig. 1d, *R*^*2*^ = 0.65). This indicates a good performance of the annual ET simulations. In addition, PML-estimated ET trend compares reasonably well to the catchment annual P–Q trend for 46 large unregulated catchments (>10,000 km^2^) distributed across North America, Europe, South America and Australia (Fig. 1b, *R*^*2*^ = 0.40). It is noted that the catchment annual P–Q trend may have noticeable errors in some catchments due to the influence of catchment storage changes.

Second, at monthly scale, the estimated ET is compared to eddy-covariance flux measurements in 95 flux towers widely spread (Fig. 1c), yielding a good agreement indicated by *R*^2^ = 0.77 and *Bias* = −6.0% (Fig. 1c). To test the robustness of the PML model for simulating ET in various climatic conditions, we split the 95 flux towers into two groups: dry and wet. The 16 sites with aridity index (the ratio of potential evapotranspiration to precipitation) more than 1.5 are deemed as ‘dry’; and the 79 sites with aridity index less than 1.5 classified as ‘wet’. Monthly estimated ET corresponds well to the measured in both dry (*R*^*2*^ = 0.77 and *Bias* = −9.7%) and wet (*R*^*2*^ = 0.77 and *Bias* = −5.3%) climatic conditions, demonstrating that the PML model performs equally well in both dry and wet conditions.

Third, the ET partitioning into its respective components is also independently evaluated. The annual E_s_ series estimated by the PML model shows good agreement with the TRMM (Tropical Rainfall Measuring Mission) soil moisture estimates (aggregated over 40 °N–40 °S TRMM coverage but excluding grid cells with over tropical forests where soil moisture retrieval is difficult), with *R*^*2*^ = 0.71 (Fig. 1e).

Fourth, the ET partitioning is also checked against 12 field experiments covering various ecosystems for which the E_s_/ET percentage ratio is obtained (Table 1). The E_s_/ET percentage ratio estimated by the PML model corresponds well to the results obtained by the field experiments, with *R*^*2*^ = 0.79.

Fifth, the annual E_i_/P percentage is also compared to measurements in 11 largely homogeneous inland forest sites (Table 2). Most sites are in humid tropical forests where E_i_ likely accounts for a significant proportion of ET. Both measurements and estimates show narrow ranges of E_i_/P percentage ratios. The measurements show that about 8–14% of annual P is partitioned to E_i_ for the selected forests, which are similar to the PML estimates that vary from 5% to 11%.

**Figure 2:**
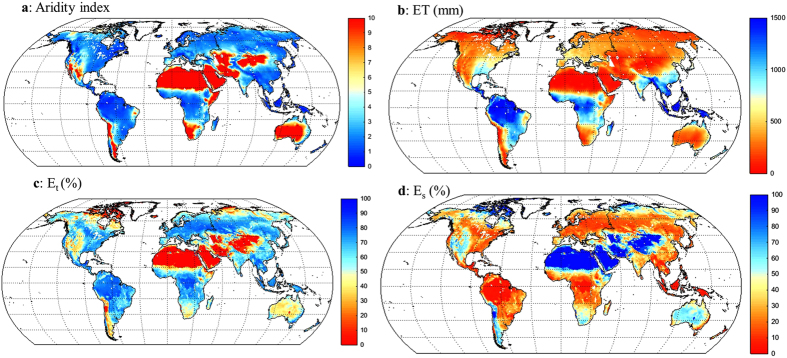
Global maps of climatology (1981–2012). (**a**) aridity index (the ratio of mean annual precipitation to mean annual potential ET). (**b**) mean annual ET. (**c**) the percentage of E_t_ to ET. (**d**) the percentage of E_s_ to ET. The maps were generated using MATLAB.

Sixth, the ET partitioning can be qualitatively evaluated in extreme climates, such as deserts in Sahara, central Asia, Arabian Peninsula, and central Australia where mean annual potential ET is more than 10 times of mean annual P (Fig. 2a). In these areas, most of precipitation is used for E_s_, as estimated by the PML model (Figs 2c and 2d).

**Figure 3:**
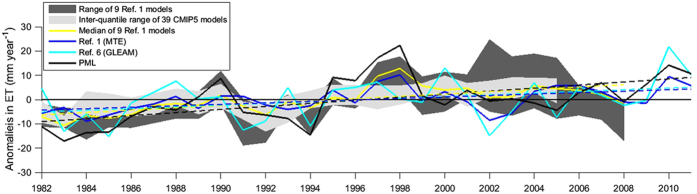
Annual global anomalies (mm year^−1^) in ET. Outputs from 39 CMIP5 models span from 1982 to 2005; outputs from 9 land surface models are from 1982 to 2008; outputs from other models (i.e., MTE, GLEAM and PML) are from 1982 to 2011. Dash lines show linear trends for the median of nine land surface models (yellow), MTE (blue), GLEAM (cyan) and PML (black), respectively.

Seventh, the ET estimates from the PML model are also compared to other two diagnostic models (MTE^1^ and GLEAM^6^), 9 land surface models^1^ and 39 Coupled Model Intercomparison Project Phase 5 (CMIP5) climate models^18^ (Fig. 3). The PML model shows reasonable agreement to the median of the nine land surface models, MTE and GLEAM in the annual global average ET estimates: *R*^*2*^ of 0.69, 0.52 and 0.31, respectively, and shows the least agreement to the median of 39 CMIP5 models: *R*^*2*^ of 0.14. All the three diagnostic models show significantly increasing ET trend over 1982–2011, being 0.68, 0.32 and 0.38 mm year^−2^ for PML (*p* < 0.01), MTE (*p* < 0.01) and GLEAM (*p* < 0.1) models, respectively.

Overall, these seven assessments, including five point to regional validations and two global evaluations, give confidence on the ET components used in our analysis.

### Global and continental summary

**Figure 4:**
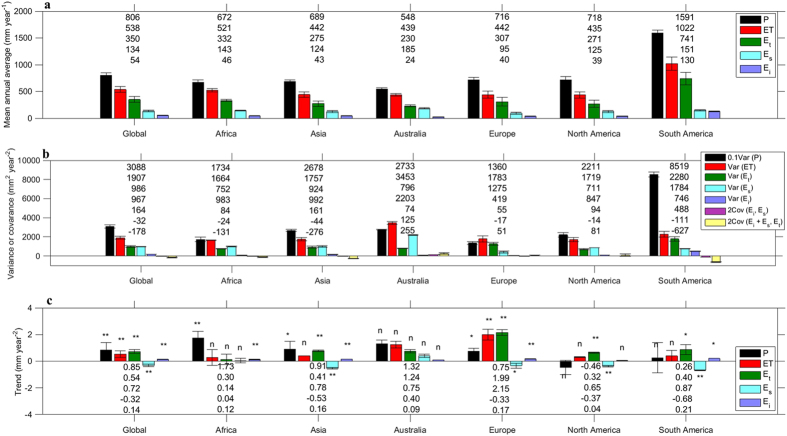
Mean global land surface and continental averages (1981–2012) of various statistics for P, ET, E_t_, E_s_ and E_i_. (**a**) Mean annual values (mm year^−1^). (**b**) Variance or covariance of annual values (mm^2^ year^−2^). (**c**) Trend (mm year^−2^). Error bars are s.d. obtained from PML simulations 1 and 2. The symbol ** indicates significance level 1–α = 99% (*p* < 0.01); the symbol * indicates significance level 1–α = 95% (*p* < 0.05); the symbol ‘n’ is not significant (*p* > 0.05). In each figure sub-part for each geographic area the numbers presented are ordered equivalently to the sub-part legend. Note that scaling is applied to the Var (P) on part (**b**).

The spatial distribution of PML mean annual ET is shown in Fig. 2b. The 1981–2012 mean ET across the global land surface (not considering water bodies and permanent ice surfaces) is 538.1 ± 56.5 mm year^−1^ (i.e., 63.2 × 10^3^ km^3^ year^−1^) (Fig. 4a) or ~67% of mean annual P (805.6 ± 41.7 mm year^−1^); in good agreement with previous estimates based on similar study periods^1^,^2^,^3^,^6^. PML E_t_ accounts for ~65% of ET (350.4 ± 54.7 mm year^−1^), E_s_ for ~25% (133.9 ± 15.0 mm year^−1^) and E_i_ for ~10% (53.7 ± 5.5 mm year^−1^). These estimates fall within the broad range of variability reported by studies based on isotopes^8^,^9^, and satellite observations and modelling^19^,^20^,^21^. The relative contribution of the different ET components varies per continent (Fig. 2c,d), reflecting water availability, energy constraints and land cover heterogeneity. Continents containing vast tracts of tropical forests, like South America, evaporate more and the relative contributions from E_t_ and E_i_ are larger (Fig. 2b,c); whereas in largely arid continents, such as Australia, E_s_ is a substantial contributor (Fig. 2d).

Averaged across the global land surface, the inter-annual variance of ET is 1907 ± 147 mm^2^ year^−2^, representing only ~6.2% of the P variance (Fig. 4b). The greater than ten-fold difference between P and ET variances is because vegetation and soil serve as ‘storage’ buffers. The E_s_ variance of 967 ± 28 mm^2^ year^−2^ is similar to the E_t_ variance globally (958 ± 94 mm^2^ year^−2^), despite the E_t_ being more than double E_s_. This is because some vegetation, especially trees, has access to deep soil water for E_t_, and have developed deep roots in response to P variability^22^,^23^. Compared to that, shallow soil water is a more immediate buffer for E_s_ responding directly to the P variability. As a result, global variance in E_s_ is comparable to that in E_t_, as E_s_ is a larger component of ET in regions with a high inter-annual variability in P, like Australia or South Africa^6^.

**Figure 5:**
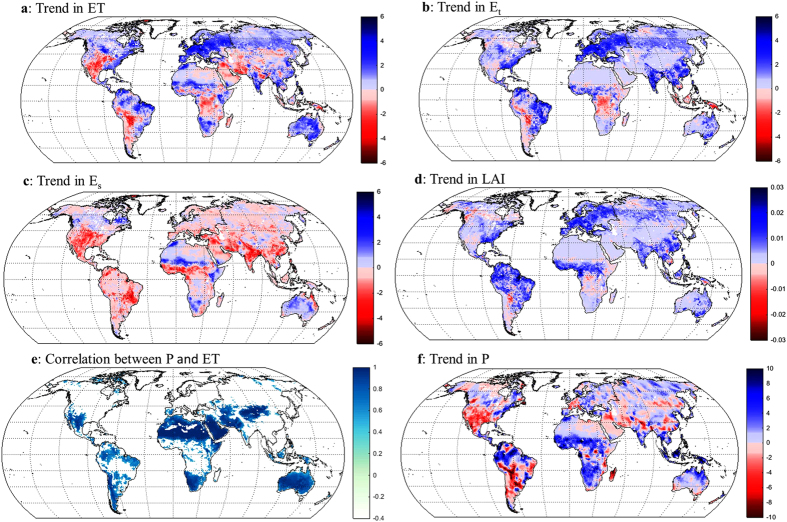
Global maps of trend and correlation (1981–2012). (**a**) ET trend (mm year^−2^). (**b**) E_t_ trend (mm year^−2^). (**c**) E_s_ trend (mm year^−2^). (**d**) LAI trend (m^2^ m^−2^ year^−1^). (**e**) correlation between annual P and annual ET (for land grid cells where *p* < 0.01, else they are white). (**f**) P trend (mm year^−2^). Trends in ET, E_t_, and E_s_ are obtained from the average of the two PML simulations. Trends in LAI are obtained from the AVHRR based LAI product, and P trends are averaged from the two P products (i.e., PGF and WFDEI). The maps were generated using MATLAB.

The global multi-decadal trend (1981–2012) in ET is positive (*p* < 0.01), i.e. in the same direction as previous estimates^1^,^6^ (Fig. 3). This is caused by significant positive trends in E_t_ (0.72 ± 0.23 mm year^−2^) and in E_i_ (0.14 ± 0.07 mm year^−2^), which are partly counter-balanced by a significant but smaller negative trend in E_s_ (–0.32 ± 0.07 mm year^−2^) (Fig. 4c). Strong positive ET trends are observed in northern and eastern Asia, India, eastern North America, Europe, northern Sub-Saharan Africa and northern and eastern Amazonia (Fig. 5a), mainly as a result of increased E_t_ (Fig. 5b). Negative ET trends are observed over parts of subtropical and temperate South America, the Middle East and western United States, and are mainly explained by reductions in E_s_ (Fig. 5a,c). Decreases in E_s_ in the Sahel^24^, Indian Subcontinent and southern China are also accompanied by increases in E_t_ (Fig. 5b,c).

### Causality analysis

The contrasting positive trend in E_t_ and negative trend in E_s_ is mostly explained by the increase in leaf area index (LAI) (Fig. 5d). This increasing trend in LAI has been attributed to CO_2_ fertilization^25^, global warming^26^, increased productivity in croplands^27^, afforestation and forest protection^27^,^28^. The increase in LAI also means more shading of the soil surface and less coupling between the atmosphere and the soil surface, and these are likely to be the main reasons for the decreasing trend in E_s_^29^,^30^.

**Figure 6:**
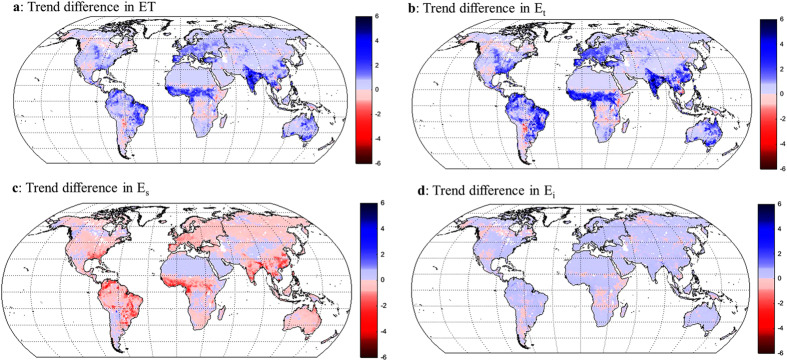
Global maps of trend difference. (**a**) ET (mm year^−2^). (**b**) E_t_ (mm year^−2^). (**c**) E_s_ (mm year^−2^). (**d**) E_i_ (mm year^−2^). Using the PML model, the trend difference is calculated between the average estimates using the observed LAI time series (experiments 1 and 2) minus the average estimates using detrended LAI time series (experiments 3 and 4); details of these experiments are provided in the Methods section. The maps were generated using MATLAB.

The trends in ET and its components due to the observed increase in LAI are further explored as the difference between PML-ET simulations obtained using the observed LAI time series and PML-ET results obtained using detrended LAI time series. The spatial distribution of the trend difference is shown in Fig. 6 and the continental summary of the trend difference is summarised in Table 3. The LAI increase causes noticeable increase in ET (Fig. 6a) in Europe, India, eastern China, eastern and northern Australia, Sahel, and eastern Amazon, which is accompanied by an increase in E_t_ (Fig. 6b) and decrease in E_s_ (Fig. 6c). The trend difference in E_i_ is much smaller compared to that in E_t_ or in E_s_ (Fig. 6d).

The contrasting trends between E_s_ and E_t_ occur mainly in croplands, grasslands, mixed forests and shrublands (see Supplementary Information Figs S1 and S2). Furthermore, the contrasting trends are confirmed when they are stratified using the LAI trend ranging from −0.025 to 0.035 m^2^ m^−2^ year^−1^ (see Supplementary Information Fig. S3).

Globally, the increase in LAI causes an increase in E_t_ and E_i_ trends by 0.71 and 0.08 mm year^−2^, and a decrease in E_s_ trend by 0.30 mm year^−2^ (Table 3). The increase in LAI causes an increase in E_t_ trend for all continents, ranging from 0.57 mm year^−2^ in Africa to 1.16 mm year^−2^ in Europe, and also causes a slight increase in E_i_ trend, ranging from 0.04 mm year^−2^ in Australia to 0.19 mm year^−2^ in Europe. In contrast, the increase in LAI reduces E_s_ trend for all continents; ranging from −0.18 mm year^−2^ in North America to −0.56 mm year^−2^ in South America. These impacts on E_t_, E_s_ and E_i_ cause noticeable ET trends increasing in all continents, ranging from 0.30 mm year^−2^ in North America to 0.84 mm year^−2^ in Europe.

**Figure 7:**
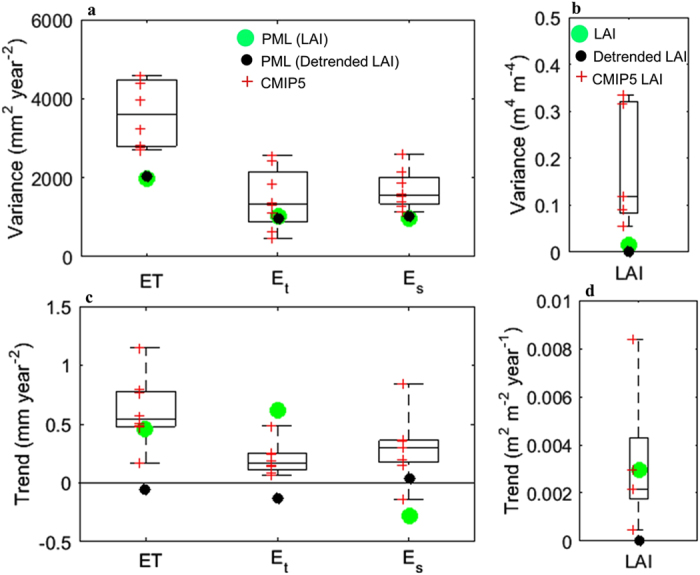
Summary of variance and trend in global ET components and LAI obtained from CMIP5 and PML models for 1981–2005. (**a**) variance in ET components (mm year^−2^). (**b**) variance in LAI (m^4^ m^−4^). (**c**) trend in ET components (mm year^−2^). (**d**) trend in LAI (m^2^ m^−2^ year^−1^). The global variance and trend in the CMIP5 and PML models are obtained using area–weighted average over all land gird cells. All eight CMIP5 models with archived ET, E_t_ and E_s_ outputs are used. Five of the eight models also archived LAI outputs. The model details are summarised in Table S1. Green dots are variance or trend estimated by PML using observed LAI time series (Fig. 7**a,c**), and are variance and trend in LAI time series (Fig. 7**b,d**); black dots are variance or trend estimated by PML using detrended LAI time series (Fig. 7**a,c**), and are variance and trend in detrended LAI time series (Fig. 7**b,d**). Red crosses are the variance or trend estimated by each CMIP5 model. The bottom, middle and top of each box are the 25^th^, 50^th^, and 75^th^ percentiles, respectively, and the bottom and top whiskers represent the minimum and maximum values.

While the trend in ET is influenced by vegetation change, the ET (and its components) variability is dominated by inter-annual climate variability^31^. Globally, this is demonstrated by Fig. 7a showing similar variance estimates in annual ET, E_t_ and E_s_ from PML using observed LAI time series and using detrended LAI time series. There is a strong correlation between P and E_s_ (and ET) (Fig. 5e) and the P trend influences E_s_ and ET trends in the southern mid-latitudes, such as the increasing trend in northern Australia and southern Africa and the decreasing trend in southern South America (Fig. 5a,c,f). In these regions, the dynamics of the EI Niño/Southern Oscillation^6^,^32^ dominate the multi-decadal P and ET variability. When stratified using the P trend, the ET trend gradually rises with increasing P trend (see Supplementary Information Fig. S4). Furthermore, a decrease in E_s_ trend and E_i_ trend occurs when the P trend decreases; a strong increase in E_i_ trend accompanies the strong increase in P trend.

## Discussion

Using the well-validated diagnostic PML model, we estimated that global ET is comprised of 65% E_t_, 25% E_s_ and 10% E_i_. Although E_t_ is larger than E_s_, their inter-annual variability is similar, because the variability in E_t_ is buffered by vegetation and soil moisture storage. The E_s_ has high inter-annual variability reflecting the high inter-annual variability of P. Regionally, the ET variability is dominated by E_t_ and E_i_ in densely vegetated and wet regions, and by E_s_ in sparsely vegetated and arid regions.

The PML model showed positive trend in ET consistent with, and close to, the median (+0.63 mm year^−2^) of the trends in four other global ET products^1^,^6^,^33^,^34^. PML and these other four products all show slightly positive ET trends globally of similar magnitude in the last three decades. Over 1981–2012, the PML model estimates positive E_t_ trend of 0.72 mm year^−2^, which is partially counteracted by a negative E_s_ trend of 0.32 mm year^−2^. These contrasting trends are primarily driven by the increasing trend in vegetation LAI.

There is a limitation in the PML model in that it does not directly account for the impact of enhanced CO_2_ concentration on vegetation water use efficiency. To quantify this potential impact, we used the CABLE^35^ global land surface model which simulates changes in water use efficiency. The experiment was performed using the same forcing data, but with two CO_2_ concentration forcings: fixed CO_2_ concentration set at 1981 level, and annual CO_2_ concentration time series from 1981 to 2012. The CABLE simulations show that the change in vegetation water use efficiency due to increasing CO_2_ concentrations from 1981 to 2012 reduces E_t_ by 0.17 mm year^−2^ and increases E_s_ by 0.04 mm year^−2^, hence reducing ET by 0.13 mm year^−2^. The positive E_t_ trend estimated by the PML model will therefore be smaller (by 20–30%) if the CO_2_ influence on vegetation water use efficiency is taken into account, but the PML result will still show the strong opposing trends in E_t_ and E_s_.

We further explore the trends in PML ET components with simulations from the eight CMIP5 models that archive outputs of ET, E_t_ and E_s_ (Fig. 7c) over a common 1981–2005 period. All eight models show positive ET trend, with the PML estimate close to the median of the trend from the eight models. However, the CMIP5 models do not show the contrasting E_t_ and E_s_ trends, and generally simulate higher positive trend in E_s_ than in E_t_. Possible reasons for this may include: (i) a more direct ET response to P in the CMIP5 models thereby implying an insufficient accounting of the vegetation and soil moisture buffering on ET in the CMIP5 models (as seen in the higher variance in ET and its components in the models (Fig. 7a)); and (ii) the limited number of CMIP5 models used here (not all models archive all ET components, and there is possible inconsistency in the definition of ET components between models). Also for 1981–2005, we compare observed LAI with simulations from the five CMIP5 models that archived LAI. Results show that the five CMIP5 models overestimate inter-annual LAI variability (Fig. 7b) and do not optimally incorporate LAI to simulate ET components (Fig. 7c) though these five CMIP5 models simulate global LAI greening reasonably (Fig. 7d). Both these findings (i.e., assessing CMIP5 ET and LAI characteristics) suggest the need for better incorporating vegetation dynamics for land-atmospheric interactions in global earth system models to adequately predict future changes in the energy and water fluxes.

## Methods

### The PML model

At each grid cell, daily ET is the sum of E_s_, E_t_ and E_i_. The PML model estimates E_s_ and E_t_ according to^10^


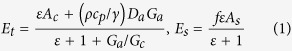


where *ε* = *s*/*γ*, in which *γ* is the psychrometric constant and *s* = de^*^/*dT* is the slope of the curve relating saturation water vapour pressure to temperature; 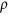
is the density of air and 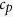 is the specific heat of air at constant pressure; 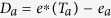 is the water vapour pressure deficit of the air (humidity deficit), in which 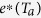 is the saturation water vapour pressure at air temperature and *e*_*a*_ is the actual water vapour pressure; *G*_*a*_ is the aerodynamic conductance; *G*_*c*_ is the canopy conductance for transpiration; and *f* is the fraction of *P* to equilibrium soil evaporation *εA*_*s*_/(1+*ε*), estimated from the accumulated precipitation over the previous month^11^. *A*, the available energy absorbed by the surface (net absorbed radiation minus soil heat flux), is partitioned using LAI into canopy absorption (*A*_*c*_) and soil absorption (*A*_*s*_). E_i_ is modelled using an adapted version of the widely adopted Gash rainfall interception model, and assumes that the ratio between the wet canopy evaporation rate and the rainfall rate does not vary between storms^36^. ET estimated at a land grid cell is aggregated from ET estimated from each land cover type within the grid cell.

There is only one-free parameter, the maximum stomatal conductance ( *g*_sx_) to calculate E_t_ in PML. The *g*_sx_ was estimated for each land cover type using the trial-and-error method by comparing (1) modelled mean annual ET with water balance ET observations (mean annual P minus mean annual Q), and (2) modelled monthly ET with *in situ* flux tower ET measurements.

At the mean annual scale (1981–2012), the PML model is further constrained by the classic Budyko framework, the Fu hydroclimatic model^37^ at each grid cell since PML is not constrained by mean annual water balances. The Fu model ensures that mean annual ET is always less than mean annual P for grid cells covered by non-crop vegetation. For cropland, mean annual ET can be larger than mean annual precipitation if irrigation uses groundwater or water transferred from other basins. Therefore, in only those grid cells covered by non-crop vegetation, the three ET components were equally scaled to match the mean annual ET (1981–2012) estimated from the Fu model. There is one parameter 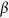 in the Fu model, which was calibrated against catchment ET observations.

### Data

Meteorological forcings from 1981 to 2012 used to drive the PML model include daily precipitation, air temperature, vapour pressure, shortwave downward radiation, longwave downward radiation and wind speed. The forcings were obtained from two widely used datasets: the Princeton Global Forcing (PGF) data^14^,^15^ and the WATCH Forcing Data ERA-Interim (WFDEI) meteorological forcing data^13^.

Vegetation forcing data were obtained as follows. LAI data from 1981 to 2011 were obtained from Boston University (BU) dataset^16^. It was derived from the Advanced Very High Resolution Radiometer (AVHRR)-NDVI data. The temporal resolution for the BU dataset is half-monthly and its spatial resolution is 0.0833°. The LAI time series data in 2011 was used for 2012. Emissivity and albedo at 0.05° spatial resolution and 8-day resolution from 1981to 2012 were obtained from the Global Land Surface Satellite (GLASS) dataset^38^. The GLASS albedo product was produced from both AVHRR (1981–1999) and Moderate Resolution Imaging Spectroradiometer (MODIS) (2000–2012) data. The GLASS longwave emissivity product was generated from both AVHRR visible and near-infrared reflectance from 1981 to 1999 and MODIS seven black-sky albedos ranging from 2000 to 2012. The surface emissivity and mean daily air temperature, was used to estimate daily outgoing longwave radiation, *R*_*lo*_. Static land cover for 16 land cover types based on the International Geosphere-Biosphere Program (IGBP) Data, generated using 2000–2001 MODIS data, were obtained from the Oak Ridge National Laboratory Distributed Active Archive Center^39^.

Validation datasets include catchment streamflow, fluxnet eddy covariance ET and over land microwave soil moisture. A total of 643 largely unregulated catchments with a widespread geographic distribution were selected to evaluate model performance at the mean annual scale. To exclude regulated catchments, major dam locations were obtained from three sources: (i) International Commission of Large Dams^40^; (ii) Meridian World Data (http://www.meridianworlddata.com/) and (iii) National Land and Water Resources Audit of Australia (http://www.nlwra.gov.au/). Daily streamflow data for the selected catchments was obtained from four sources: (i) the Global Runoff Data Centre (located in Germany, http://www.bafg.de/GRDC/EN/Home/homepage_node.html); (ii) the Water Information Research and Development Alliance between CSIRO and Australian Bureau of Meteorology^41^; (iii) the Model Parameter Estimation Experiment (MOPEX)^42^ and (iv) the Chinese Academy of Sciences. Each catchment had at least 5 years of observations. Catchment mean annual ET values were estimated as mean annual P minus mean annual runoff (Q), assuming that changes in soil water storage are negligible in the long term^43^.

A total of 95 fluxnet towers were selected to evaluate model performance at monthly scale. Data for the 93 towers were obtained from the LaThuile FLUXNET dataset. An additional two sites were obtained, one from the OzFlux and another from AmeriFlux. The selected sites span a wide range of climate regimes, covering a total of 11 vegetation types^39^. These include: grasslands (GRA), evergreen broadleaf forest (EBF), croplands (CRO), mixed forest (MF), evergreen needleleaf forest (ENF), wetlands (WET), open shrublands (OSH), deciduous broadleaf forest (DBF), savannas (SAV), woody savannas (WSA), and closed shrublands (CSH).

Each flux site meets the following criteria: (1) mostly homogeneous land cover at 1 km radius from the flux tower (checked with Google Earth); (2) daily energy balance closure of more than 75%; and (3) more than 2 years of daily data (during days with no precipitation) available. Note here that our evaluation compares the ET_PML_ at 0.50° spatial resolution (i.e., the resolution of the global forcing data) against ET_flux_ representing ET from a radius of tens to hundreds of metres (depending on biophysical, atmospheric and instrumental characteristics).

Annual variation of ET and its components was validated against that of observed soil moisture in sparsely-vegetated regions. The observed soil moisture data were obtained from the radiometer Microwave Instrument on board NASA’s Tropical Rainfall Measuring Mission (TRMM) that started providing passive microwave observations at 10.7 GHz (X-band) and eight higher frequencies including the 37 GHz (Ka) band from December 1997. The observations can be assimilated in a microwave radiation transfer model to infer soil moisture, soil and canopy temperature and vegetation optical depth. We used the top soil moisture retrieved from the Land Parameter Retrieval Model^44^ based on L- and Ka-band brightness temperatures. The retrieved soil moisture represents the top few centimeters corresponding to 10.7 GHz (X-band). The platform TRMM covers regions between 40 °N and 40 °S. Due to the influence of dense vegetation, reasonable soil moisture retrievals over tropical forests are not available and thus masked out^45^. The dataset used in this study was resampled to 0.50° spatial resolution and aggregated to monthly average for January 1998 to December 2012.

### Modelling experiments

The PML model simulations used two forcing datasets (PGF and WFDEI). Four simulations were run as follows: (1)PML + PGF; (2) PML + WFDEI; (3) PML + PGF (detrended LAI); (4) PML + WFDEM (detrended LAI).

Simulations 1–2 were carried out using observed LAI time series, and simulations 3–4 were carried out by repeating the simulations 1–2 but using detrended LAI (i.e. removing the long-term trend, but allowing for sub-annual variation related to seasonal cycles). The difference between simulations 1–2 and simulations 3–4 is used to quantify the impacts of LAI change on trends and variability in ET and its components.

### Statistical analysis

Annual variance in ET at each grid cell was partitioned into E_t_, E_s_ and E_i_ components, and expressed as 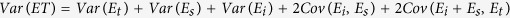. The Mann–Kendall Tau-b non-parametric test including Sen’s slope method^46^ was used for trend analysis and significance testing.

## Additional Information

**How to cite this article**: Zhang, Y. *et al*. Multi-decadal trends in global terrestrial evapotranspiration and its components. *Sci. Rep*. **6**, 19124; doi: 10.1038/srep19124 (2016).

## Supplementary Material

Supplementary Information

## Figures and Tables

**Table 1:** Comparing the estimated (average from simulations 1 and 2) percentage of soil evaporation (E_s_) corresponding to total evapotranspiration (ET) to the measured percentage obtained from 12 field experiments.

Site	Lat.	Long.	*E*_*s*_*/ET (Measured)*	*E*_*s*_*/ET (Estimated)*	Land cover type	Reference	Year length
Santa Rita Experimental Range (USA)	31.91	−110.84	58	42	Shrub	Cavanaugh *et al*. (2011)^47^	1
Walnut Gulch Experimental Watershed (USA)	31.74	−110.05	53	54	Shrub	Cavanaugh *et al*. (2011)^47^	1
Oak Ridge (USA)	35.96	−84.29	16	16	Forest	Wilson (2001)^48^	3
Steinkreuz catchment (Germany)	49.87	10.47	10	20	Forest	Kostner (2011)^49^	5
Luan Cheng (China)	37.88	114.68	30	28	Cropland	Liu *et al*. (2002)^50^	5
Oerst Forests (New Zealand)	−42.22	172.25	10	9	Forest	Kelliher *et al*. (1992)^51^	1
Sultana Vineyard (Australia)	−34.22	142.03	43	46	Vineyard	Yunusa *et al*. (2004)^52^	2
Punjab Agricultural University (India)	30.93	75.87	39	23	Cropland	Balwinder *et al*. (2011)^53^	2
Yang Ling (China)	34.33	108.40	30	21	Cropland	Kang *et al*. (2003)^54^	10
lower coastal plain, North Carolina (USA)	35.80	−76.67	14	24	Forest	Domec *et al*. (2012)^55^	3
Norunda Common (Sweden)	60.08	17.05	13	16	Forest	Constaintin *et al*. (1999)^56^	2
Menglun Forest Reserve, Yunnan (China)	21.93	101.27	4	6	Tropical Forest	Liu *et al*. (2006)^57^	2

**Table 2:** Comparing the estimated (average from simulations 1 and 2) percentage of interception evaporation (E_i_) corresponding to annual precipitation (P) to the measured obtained from 11 field experiments.

Site	Lat.	Lon.	E_i_/P (Measured)	E_i_/P (Estimated)	Land cover Type	Reference	Year length
Tapajos National Forest south of Santarém (Brazil)	−2.9	−54.9	11.6	9.89	Forest	Czikowsky and Fitzjarrald (2009)^58^	3
Lambir Hills National Park, Sarawak (Malaysia)	4.3	114.0	8	9.79	Forest	Kume *et al*. (2011)^59^	10
Central Kalimantan (Indonesia)	−1.30	112.38	11.4	9.96	Forest	Asdak *et al*. (1998)^60^	1
Pena Rojo (Colombia)	−0.62	−70.72	12	9.93	Forest	Martin *et al*. (2000)^61^	3
Reserva Florestal Ducke (Brazil)	−2.95	−59.95	8.9	10.47	Forest	Lloyd and Marques (1988)^62^	2
Abracos forests (Brazil)	−10.1	−61.9	11.6	9.86	Forest	Ubarana (1996)^63^	2
Cuieiras Biological Reservation (Brazil)	−2.5	−60.2	13.3	10.18	Forest	Cuartas *et al*. (2007)^64^	2
Central Amazonia (Brazil)	−2.95	−59.95	9.1	10.47	Forest	Shuttleworth (1988)^65^	2
Tai National Park (Ivory Coast)	5.85	−7.34	9.2	9.21	Forest	Hutjes *et al*. (1990)^66^	1
Central Kalimantan (Indonesia)	0.1	113.9	13	9.40	Forest	Vernimmen *et al*. (2007)^67^	1
South−east of Lisbon (Portugal)	38.63	−8.6	10.8	5.61	Forest	Valente *et al*. (1997)^68^	3

**Table 3:** Trends (mm year^−2^) in ET and its components (average from simulations 1 and 2) obtained using observed LAI time series and those obtained (average from simulations 3 and 4) using the detrended LAI across globe and each continent.

	LAI inputs	Global	Africa	Asia	Australia	Europe	North America	South America
ET	Trended	0.54^**^	0.30^n^	0.41^n^	1.24^n^	1.99^**^	0.32^n^	0.40^n^
	Detrended	0.05^n^	−0.08^n^	−0.05^n^	0.55^n^	1.15^**^	0.02^n^	−0.29^n^
	Difference	0.49	0.38	0.46	0.69	0.84	0.30	0.69
E_t_	Trended	0.72^**^	0.14^n^	0.78^**^	0.75^n^	2.15^**^	0.65^**^	0.87^*^
	Detrended	0.01^n^	−0.44^*^	0.14^n^	−0.15^**^	0.99^**^	0.22^n^	−0.22^n^
	Difference	0.71	0.57	0.64	0.90	1.16	0.43	1.09
E_s_	Trended	−0.32^**^	0.04^n^	−0.53^**^	0.40^n^	−0.33^*^	−0.37^**^	−0.68^**^
	Detrended	−0.02^n^	0.28^n^	−0.28^*^	0.66 ^n^	0.17^n^	−0.19^n^	−0.12^n^
	Difference	−0.30	−0.24	−0.25	−0.26	−0.50	−0.18	−0.56
E_i_	Trended	0.14^**^	0.12^**^	0.16^**^	0.09 ^n^	0.17^**^	0.04^n^	0.21^*^
	Detrended	0.06^n^	0.07^n^	0.10^*^	0.05^n^	−0.02^n^	−0.01^n^	0.05^n^
	Difference	0.08	0.05	0.06	0.04	0.19	0.05	0.15

The symbols ^**^, ^*^ and ^n^ denote significance level 1–α = 99% (*p* < 0.01), 1–α = 95% (*p* < 0.05), and not significant (*p* > 0.05), respectively. The “Difference” is calculated as “Trended” minus “Detrended”.
