# Supplementary material for: Multi-decadal trends in global terrestrial evapotranspiration and its components
Source: Sci Rep. 2016 Jan 11;6:19124. doi: 10.1038/srep19124 (PMC4707530; doi:10.1038/srep19124)
Supplement: Supplementary Information [file srep19124-s1.pdf]

SUPPLEMENTARY INFORMATION

Multi-decadal trends in global terrestrial evapotranspiration and its components

Yongqiang Zhang[1*], Jorge L. Peña-Arancibia[1], Tim R. McVicar[1,2], Francis H.S. Chiew[1], Jai Vaze[1], Changming Liu[3], Xingjie Lu[4], Hongxing Zheng[1], Yingping Wang[4], Yi Y. Liu[2], Diego Miralles[5,6], Ming Pan[7]

[1]*CSIRO Land and Water, GPO Box 1666, Canberra ACT 2601, Australia*

[2]*ARC Centre of Excellence for Climate System Science & Climate Change Research Centre, University of New South Wales, Sydney 2052, Australia*

[3]*Institute of Geographic Sciences and Natural Resources Research, Chinese Academy of Sciences, Beijing 100101, China*

[4]*CSIRO Ocean and Atmosphere, PMB #1, Aspendale, Victoria 3195, Australia*

[5]*Department of Earth Sciences, VU University Amsterdam, Amsterdam 1081 HV, The Netherlands*

[6]*Laboratory of Hydrology and Water Management, Department of Forest and Water Management, Ghent University, B-9000 Ghent, Belgium*

[7]*Department of Civil and Environmental Engineering, Princeton University, NJ 08544, U.S.A.*

[*]Corresponding author:

Yongqiang Zhang

CSIRO Land and Water, Clunies Ross Street, Canberra 2601, Australia

E-mail: yongqiang.zhang@csiro.au

Tel.: +61 2 6246 5761

Fax: +61 2 6246 5800

This file includes three sections:

1. Summary of CMIP5 models;
2. Stratifying land area using land cover types; and
3. Stratifying trend in ET and its components using P and LAI trends.

1. THE CMIP5 MODELS

Table S1 summarises eight CMIP5 models with archived evapotranspiration (ET), soil evaporation (E_s) and vegetation transpiration (E_t). The trend and variance estimated from the eight CMIP5 models are compared to those obtained from the PML model. Five of the eight CMIP5 models archived Leaf Area Index (LAI).

Table S1 | Eight CMIP5 models with archived evapotranspiration (ET), soil evaporation (E_s) and vegetation transpiration (E_t)

Modeling Center (or Group)	Institute ID	Model Name	Vegetation variability	LAI output
Beijing Climate Center, China Meteorological Administration	BCC	BCC-CSM1.1(m)	Yes	Yes
College of Global Change and Earth System Science, Beijing Normal University	GCESS	BNU-ESM	Yes	Yes
Canadian Centre for Climate Modelling and Analysis	CCCMA	CanESM2	Yes	Yes
Atmosphere and Ocean Research Institute (The University of Tokyo), National Institute for Environmental Studies, and Japan Agency for Marine-Earth Science and Technology	MIROC	MIROC4h	No	No
		MIROC5	No	No
Japan Agency for Marine-Earth Science and Technology, Atmosphere and Ocean Research Institute (The University of Tokyo), and National Institute for Environmental Studies	MIROC	MIROC-ESM	Yes	Yes
		MIROC-ESM-CHEM	Yes	Yes
Meteorological Research Institute	MRI	MRI-CGCM3	Yes	No

2. STRATIFYING LAND AREA USING LAND COVER TYPES

We further explore the influence of land cover types[1] to stratify the land area where LAI shows the co-trends to E_t and the opposing trends to E_s (Figures S1 and S2). Among the 9 major land cover types, mixed forests, croplands, shrublands and evergreen forests are four top contributors for the co-positive trend between LAI and E_t. The four top contributors for the opposing trends, i.e., the negative E_s accompanied by the positive E_t, are evergreen forests, shrublands, croplands and mixed forests. The increased trend in LAI for croplands may be caused by multiple factors, such as the application of fertilizers and intensification of irrigation to increase crop yields, increased temperature[2], and also increased atmosphere CO_2 concentration[3]. In mixed forests, it seems that afforestation (either by natural regeneration or by plantation forestry, or the establishment of new forest in areas where there were none before), woody encroachment in drylands and forest protection play an important role for the greening trend[3,4].

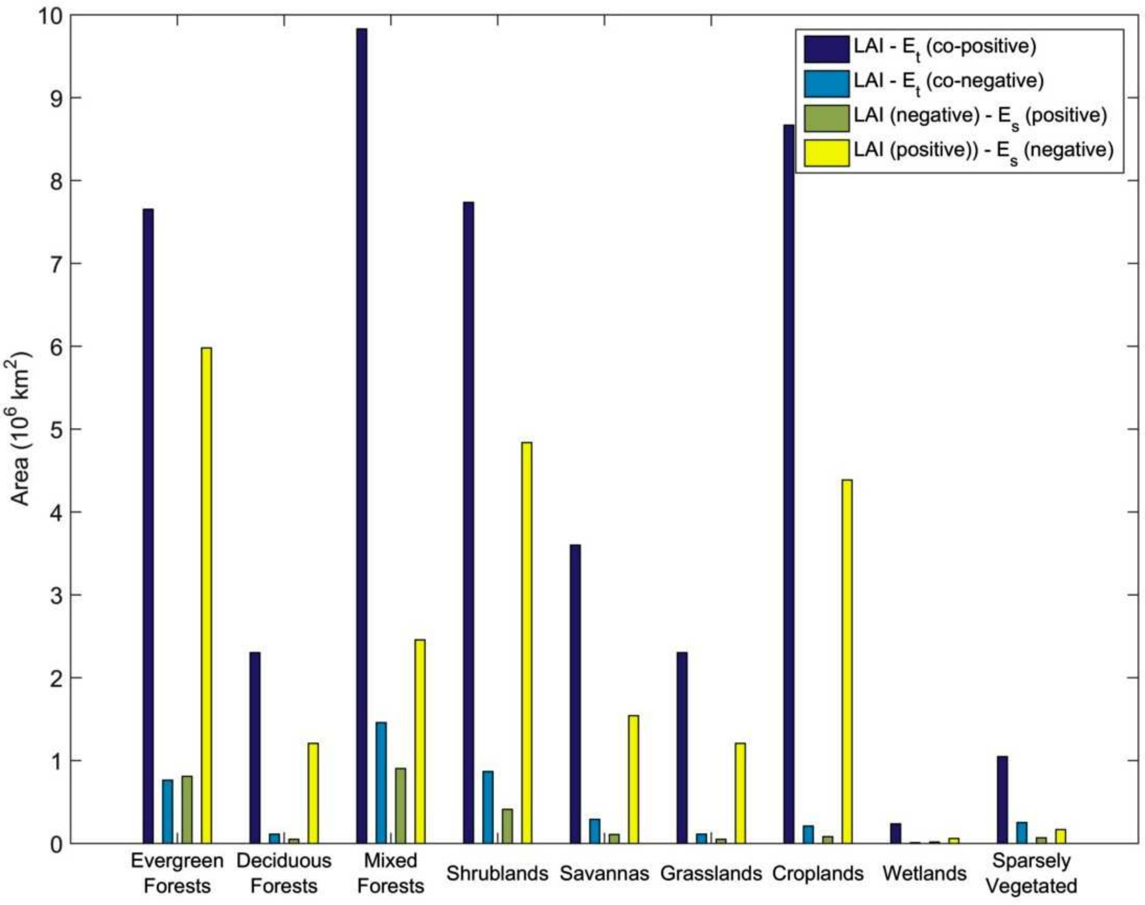

Figure S1 | Stratifying land area for the co-positive or co-negative trend ($p < 0.1$) between LAI and E_t and the opposing trend ($p < 0.1$) between LAI and E_s using IGBP land cover types. Evergreen needleleaf forest and Evergreen broadleaf forest are grouped into evergreen forests; deciduous needleleaf forest and deciduous broadleaf forest are grouped into deciduous forests; closed shrublands and open shrublands are grouped into shrublands; woody savannas are considered as savanna. E_t and E_s are estimated from the Princeton Global Forcing data. Calculation of total land area does not include deserts (LAI→0): Sahara, Arabian Peninsula and central Asia, water bodies and permanent ice surfaces.

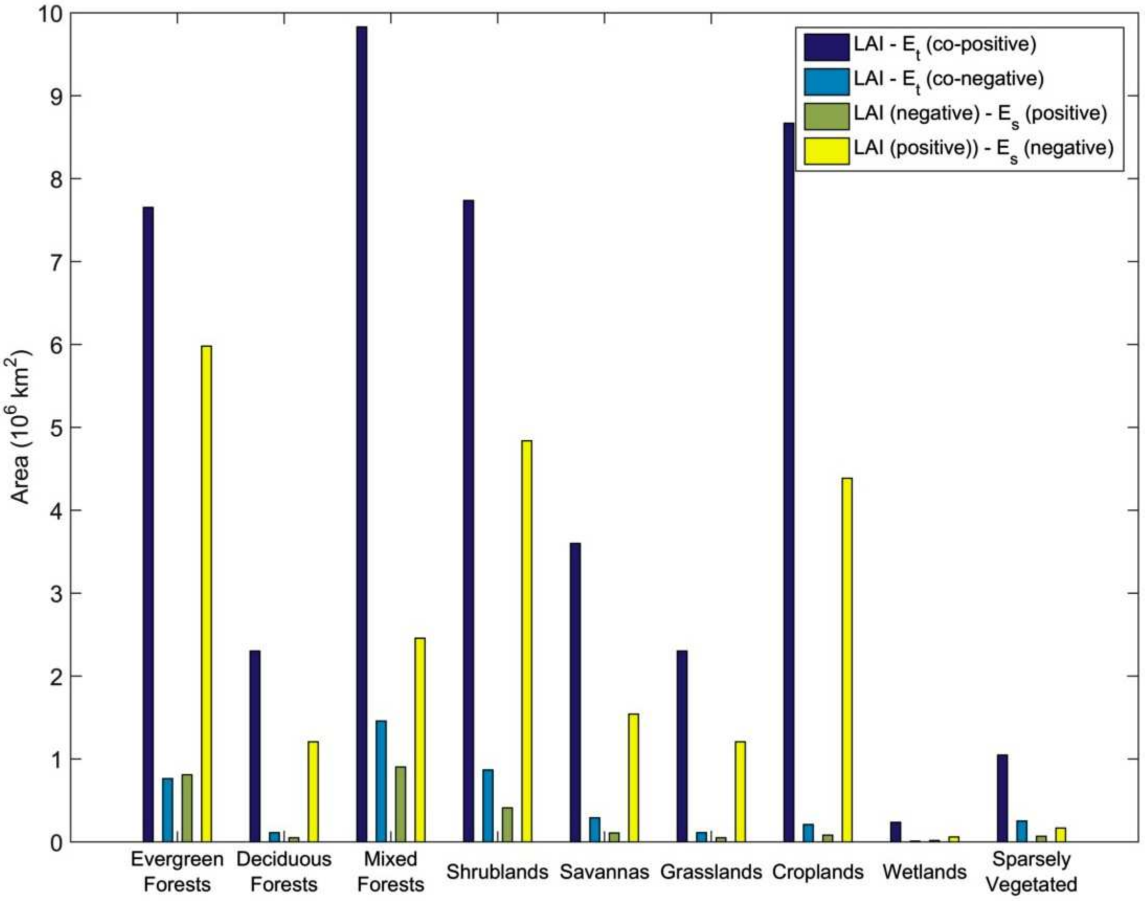

Figure S2 | Same as for Figure S1, but E_t and E_s are estimated from the WFDEI forcing data.

3. STRATIFYING TREND IN ET AND ITS COMPONENTS USING P AND LAI TRENDS

Figure S3 summarises the trend in ET and its components (E_t, E_s and E_i (evaporation from intercepted rainfall from vegetation)), stratified using the LAI trend. Most of the ET trends are negative with the LAI trend less than -0.005 m^2 m^{-2} $year^{-1}$, and most of the ET trends are positive with the LAI trend more than 0.005 m^2 m^{-2} $year^{-1}$. Furthermore, the ET trend gradually increases along the LAI trend bands varying from -0.025 to 0.035 m^2 m^{-2} $year^{-1}$. The E_s trend is complementary to the E_t trend, i.e. E_t trend increasing and E_s trend decreasing with increasing LAI trend bands. The increasing trend in E_t is about twofold of the decreasing trend in E_s. Compared to E_s and E_t, E_i shows much less variability in its trend (when compared across LAI trend bands). Similar to the E_t trend, the E_i trend is also gradually increasing along the LAI trend bands.

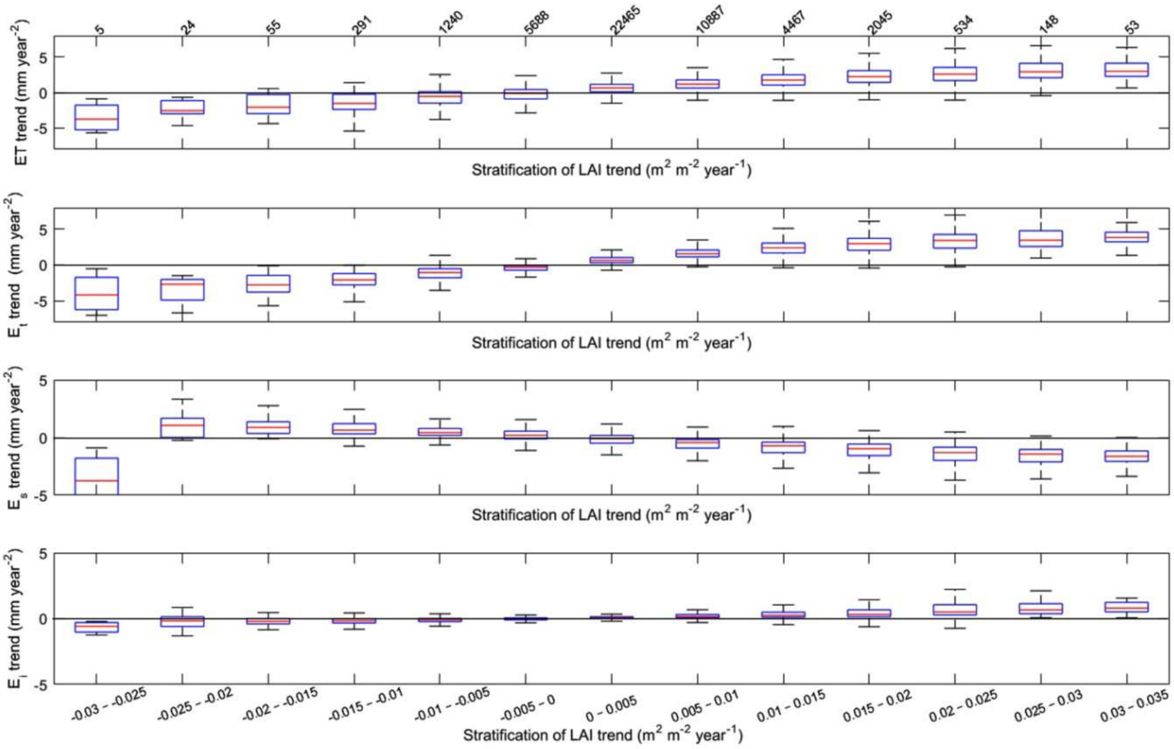

Figure S3 | Stratifying trend in ET and its components using leaf area index (LAI) trend. Number above the top panel is the number of grid cells within each LAI trend band. In each boxplot, bottom, middle, and top are the 25th, 50th, and 75th percentiles, and bottom and top whiskers are the 10th and 90th percentiles.

Figure S4 summarises the trend in ET and its components (E_t, E_s and E_i) in each precipitation (P) trend band. Most of the ET trends are negative with the P trend less than -4.0 mm year^{-2}, and most of the ET trends are positive with the P trend more than 4.0 mm year^{-2}. Furthermore, the ET trend gradually rises along the P trend bands varying from -18 to 28 mm year^{-2}. The strong decrease in E_s and E_i takes place when P shows the strong decrease; the strong increase in E_i accompanies the strong increase in P.

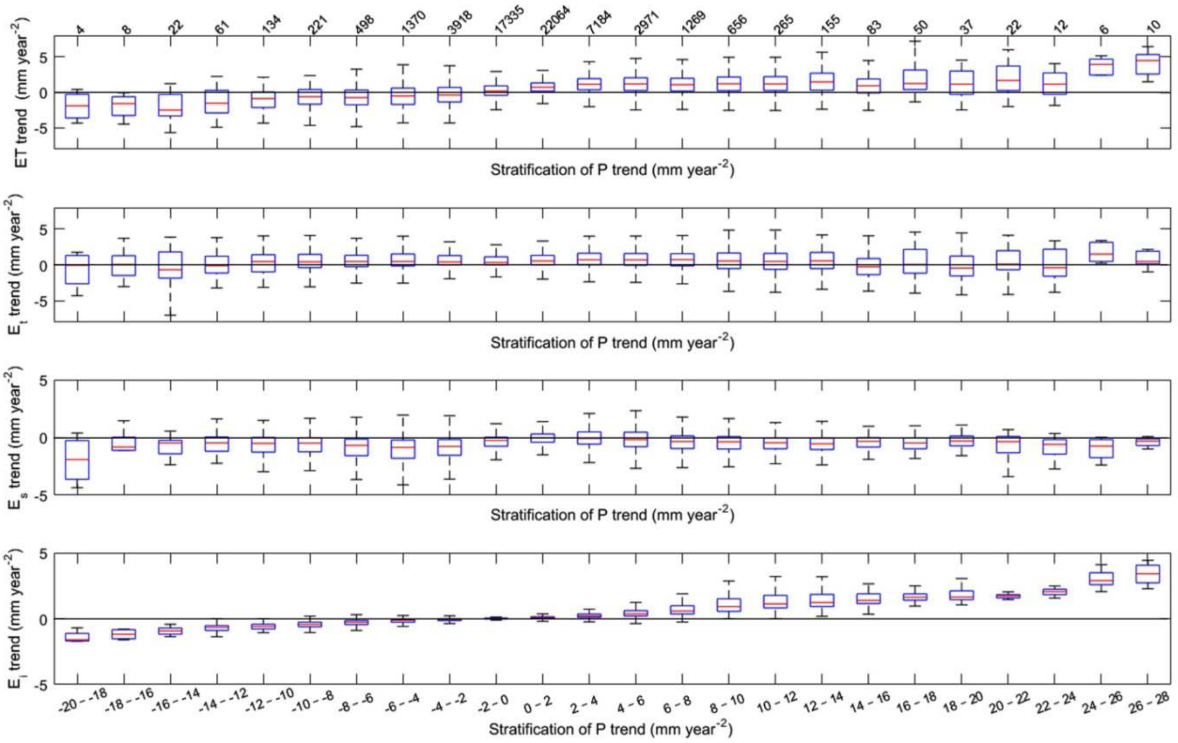

Figure S4 | Stratifying trend in ET and its components using precipitation (P) trend. Number above the top panel is the number of grid cells within each P trend band. In each boxplot, bottom, middle, and top are the 25th, 50th, and 75th percentiles, and bottom and top whiskers are the 10th and 90th percentiles.

References

1 Friedl, M. A., A.H. Strahler, and J. Hodges. ISLSCP II MODIS (Collection 4) IGBP Land Cover, 2000-2001. In Hall, Forest G., G. Collatz, B. Meeson, S. Los, E. Brown de Colstoun, and D. Landis (eds.). ISLSCP Initiative II Collection. Data set. Available on-line [http://daac.ornl.gov/] from Oak Ridge National Laboratory Distributed Active Archive Center, Oak Ridge, Tennessee, U.S.A. doi:10.3334/ORNLDAAC/968 (2010). (Date of access: 22/01/2014)

2 Xiao, J. F. & Moody, A. Trends in vegetation activity and their climatic correlates: China 1982 to 1998. *Int. J. Remote Sens.* **25**, 5669-5689, doi:10.1080/01431160410001735094 (2004).

3 Donohue, R. J., Roderick, M. L., McVicar, T. R. & Farquhar, G. D. Impact of CO_2 fertilization on maximum foliage cover across the globe's warm, arid environments. *Geophysical Research Letters* **40**, 3031-3035, doi:10.1002/grl.50563 (2013).

4 Dardel, C. *et al.* Re-greening Sahel: 30 years of remote sensing data and field observations (Mali, Niger). *Remote Sensing of Environment* **140**, 350-364, doi:10.1016/j.rse.2013.09.011 (2014).
